# Supplementary material for: The Mitotic Spindle in the One-Cell C. elegans Embryo Is Positioned with High Precision and Stability
Source: Biophys J. 2016 Oct 18;111(8):1773–84. doi: 10.1016/j.bpj.2016.09.007 (PMC5071606; doi:10.1016/j.bpj.2016.09.007)
Supplement: Document S1. Figs. S1 and S2 [file mmc1.pdf]

Biophysical Journal, Volume *111*

Supplemental Information

The Mitotic Spindle in the One-Cell *C. elegans* Embryo Is Positioned with High Precision and Stability

Jacques Pécréaux, Stefanie Redemann, Zahraa Alayan, Benjamin Mercat, Sylvain Pastezeur, Carlos Garzon-Coral, Anthony A. Hyman, and Jonathon Howard

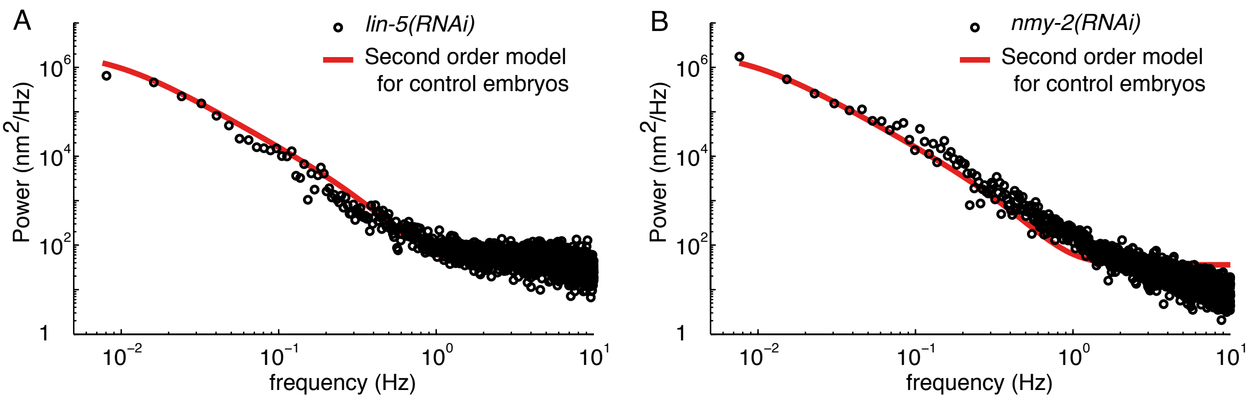

Figure S1, related to Figure 3: Spindle positioning in *lin-5(RNAi)* and *nmy-2(RNAi)* embryos

A Average power spectra of the transverse spindle position for six *lin-5(RNAi)* embryos (black open circles). For comparison, the second-order model fit to the control embryos (From Figure 2) is shown in red.

B Average power spectra of the transverse spindle position for seven *nmy-2RNAi)* embryos (black open circles). For comparison, the second-order model fit to the control embryos (From Figure 2) is shown in red.

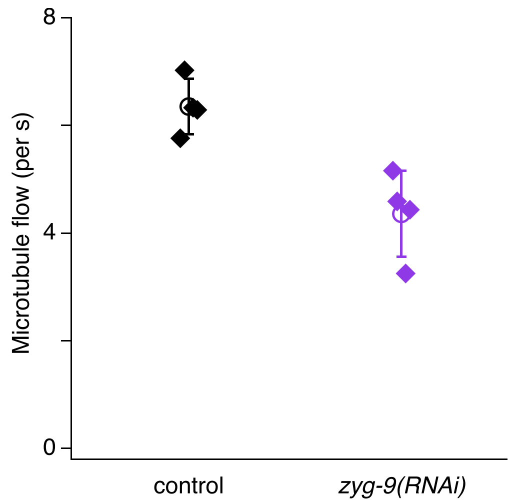

Figure S2, related to Figure 3: Rate of arrival of microtubule ends at the cortex in control and *zyg-9(RNAi)* embryos.

Microtubules ends in a YFP::α-tubulin strain were imaged at the cortex by spinning disk microscopy as described in (23). The observed cortical area was ~1200 µm^2. The number of microtubules tracks was divided by the observation time to obtain the arrival rate. Results from four *zyg-9(RNAi)* embryos (purple) are compared to results from four controls (black).
